# Short-term outcomes of ultrasound-assisted catheter-directed thrombolysis and mechanical thrombectomy in management of intermediate-risk pulmonary embolism

**DOI:** 10.1016/j.jvsv.2026.102489

**Published:** 2026-03-20

**Authors:** Crista E. Horton, Matthew S. Wi, Matthew Sayegh, Douglas Mancuso, Lauren A. Huntress, Joseph A. Savarese, John J. Ricotta, Joseph J. Ricotta

**Affiliations:** a Florida Atlantic University Charles E. Schmidt College of Medicine, Boca Raton, FL; b Department of Vascular Surgery, Delray Medical Center, Delray Beach, FL

**Keywords:** Aspiration thrombectomy, Intermediate-risk pulmonary embolism, Mechanical thrombectomy, Submassive pulmonary embolism, Ultrasound-assisted catheter-directed thrombolysis

## Abstract

**Objective:**

The aim of this study was to compare preoperative right ventricle-to-left ventricle (RV/LV) ratios by computed tomography angiography (CTA) and echocardiography and evaluate treatment outcomes of ultrasound-assisted catheter-directed thrombolysis (USAT) or mechanical thrombectomy (MT) in patients with intermediate-risk pulmonary embolism (PE).

**Methods:**

We retrospectively identified patients treated for intermediate-risk PE from 2018 to 2023 defined by systolic blood pressure (BP) >90 mmHg and right ventricle dysfunction defined by RV/LV ratio >0.9 via echocardiogram. RV/LV ratios were also evaluated on diagnostic CTA for comparison to echocardiogram values. Patients underwent USAT or MT based on thrombus location and contraindications to thrombolysis. Primary outcomes were intensive care unit (ICU) length of stay (LOS), hospital LOS, and 30-day and 1-year survival. Secondary outcomes included RV/LV change post-treatment on echocardiography, concomitant deep vein thrombosis (DVT), elevated cardiac biomarkers, blood transfusion, history of DVT/PE or malignancy, and imaging modality comparison in determination of RV/LV and RV/LV changes.

**Results:**

Of 120 patients from a single center treated for intermediate-risk PE, 100 underwent USAT and 20 MT. There was no difference in ICU LOS (2.4 vs 2.6 days; *P* = .67) or hospital LOS (4.8 vs 5.7 days; *P* = .41) for USAT and MT, respectively. There was no difference in 30-day (98% vs 100%), or 1-year survival (96% vs 90%). MT patients had greater incidence of blood transfusion (25% vs 5%; *P* = .011). CTA overestimated RV/LV ratios preoperatively compared with echocardiography (1.4; SD, 0.4 vs 1.3; SD, 0.2; *P* = .027). Patients undergoing MT were more likely to have identified lower extremity DVT compared with patients undergoing USAT (90% vs 65%; *P* = .033). Other secondary outcomes showed no significant differences between treatment groups.

**Conclusions:**

Both USAT and MT provide excellent short-term and intermediate outcomes in patients with intermediate-risk PE, similar survival rates, similar post-treatment change in right heart dysfunction, and comparable ICU and hospital stays. MT is associated with higher transfusion rates, so caution should be exercised in patients felt to be at risk from further blood loss. These findings support individualized, anatomy- and risk-based selection of catheter-based therapy within a multidisciplinary PE response framework.

**Article Highlights**

• **Type of Research:** Single-center retrospective cohort study
• **Key Findings:** Intermediate-risk pulmonary embolism treated with ultrasound-assisted catheter-directed thrombolysis or mechanical thrombectomy showed similar short-term outcomes, including survival, hospital stay, and intensive care unit stay. Both treatments produced comparable improvement in right-heart function as determined by postoperative echocardiography. Preoperative computed tomography angiography overestimated right ventricle-to-left ventricle ratios compared with preoperative echocardiography.
• **Take Home Message:** Ultrasound-assisted catheter-directed thrombolysis and mechanical thrombectomy are excellent surgical options with comparable short-term success rates for intermediate-risk pulmonary embolism.

Pulmonary embolism (PE) remains a leading cause of cardiovascular morbidity and mortality worldwide, with clinical presentations spanning from low-risk to high-risk disease.^1^, ^2^, ^3^ Patients with low-risk PE are hemodynamically stable and lack evidence of right ventricular (RV) dysfunction, whereas those with high-risk (massive) PE present with systemic hypotension, shock, or cardiac arrest.^1^^,^^4^ Between these extremes lies intermediate-risk (submassive) PE—a heterogeneous population characterized by hemodynamic stability accompanied by objective evidence of RV strain or myocardial injury.^3^^,^^5^^,^^6^ This subgroup represents a therapeutic gray zone, as patients are at increased risk for adverse outcomes, yet do not uniformly benefit from systemic thrombolysis due to risk of bleeding. Recent professional society guidelines, including the 2026 American Heart Association/American College of Cardiology Multisociety Guidelines for Acute Pulmonary Embolism, underscore the lack of consensus on optimal management of intermediate-risk PE and highlight the need for further data on this topic.^7^

RV dysfunction is central to the pathophysiology and prognosis of intermediate-risk PE, a component of which is determined by RV to left ventricle (RV/LV) ratio. RV/LV ratio is calculated by measuring the maximal diameter of both ventricular chambers in the apical 4-chamber view at end-diastole (on echocardiography) or the standard axial view (on computed tomography angiography [CTA]) perpendicular to the long axis of the heart, and dividing RV diameter by LV diameter.^8^^,^^9^ An elevated RV/LV ratio has been defined as >0.9 and is consistently associated with increased morbidity and mortality.^10^ CTA is widely used for PE diagnosis and provides rapid assessment of clot burden and RV dilation; however, it offers a static, two-dimensional, anatomical assessment and has the potential to overestimate RV strain. In contrast, echocardiography allows dynamic, real-time evaluation of RV function and is more specific for risk stratification.^11^ Consequently, we believe echocardiography plays a pivotal role in determining the need for escalation beyond anticoagulation in patients with intermediate-risk PE.

Systemic thrombolysis can reduce RV afterload and improve hemodynamics, but it has not demonstrated a clear mortality benefit in intermediate-risk PE and carries significant risk of major bleeding.^12^^,^^13^ As a result, endovascular therapies have gained increasing attention as alternatives that aim to rapidly relieve RV strain while minimizing hemorrhagic complications by limiting or eliminating the use of thrombolytic agents. These approaches include catheter-directed thrombolysis, particularly ultrasound-assisted catheter-directed thrombolysis (USAT), which delivers low-dose thrombolytic therapy directly into the thrombus, and mechanical thrombectomy (MT), which achieves clot removal with little or no use of lytic agents.^14^^,^^15^

Prospective and randomized studies have shown that both USAT and MT lead to significant reductions in RV/LV ratio and improvement in RV function in intermediate-risk PE.^16^, ^17^, ^18^, ^19^, ^20^, ^21^, ^22^ However, direct comparisons between these two strategies remain limited, particularly with respect to early RV recovery, bleeding risk, resource utilization, and short- and intermediate-term clinical outcomes. Moreover, the optimal integration of these modalities into modern treatment algorithms—especially within Pulmonary Embolism Response Team (PERT)-driven care—remains incompletely defined.

The objective of this study was to compare USAT and MT in patients with intermediate-risk PE managed by a multidisciplinary PERT. We sought to evaluate effectiveness of each modality in reversing RV dysfunction, as measured by changes in RV/LV ratio on echocardiography, and to assess clinically meaningful outcomes including intensive care unit (ICU) length of stay (LOS) and hospital length of stay (HLOS), and 30-day and 1-year survival. By analyzing 120 consecutive patients treated at our institution over a 5-year period, we aim to provide practical insights, inform procedural selection, and optimize care for this complex patient population.

## Methods

### Study design

This is a retrospective cohort study including 120 consecutive adult patients ≥18 years of age including patients who were identified as having intermediate-risk PE. All patients identified by a radiologist as having a PE with RV/LV ratio >0.9 by non-gated CTA underwent PE response team activation. Patients with RV/LV ratio >0.9 by echocardiography were taken for catheter-based intervention. All patients underwent one of two treatment interventions: USAT (EKOS; Boston Scientific) (n = 100) or MT (n = 20) at a single institution between February 2018 through December 2023. Mechanical thrombectomy was performed with either 12-French or 16-French large-bore MT catheters with computer-assisted aspiration (Penumbra Indigo Lightning System). Intermediate-risk PE was defined by the following criteria: (1) Diagnosis of PE on imaging with hemodynamic stability with systolic blood pressures >90 mmHg, no tachycardia, or hypoxia; (2) RV dysfunction or myocardial necrosis, as defined by RV dilation or elevated cardiac biomarkers including troponin and/or brain natriuretic peptide (BNP). RV dysfunction was assessed by apical 4-chamber right ventricle diameter divided by left ventricle diameter >0.9, as seen on echocardiogram and CTA. Echocardiography when available was deemed definitive in detecting right heart strain. We utilized radiology reports in determination of RV/LV ratios. All patients diagnosed with intermediate-risk PE meeting these criteria underwent either USAT or MT. MT was performed when the majority of thrombus was centrally located (main pulmonary artery trunks) or there was a contraindication to lytic therapy, whereas USAT was employed in all other eligible patients.

During USAT, the catheter was advanced into the bilateral main pulmonary arteries and positioned within the thrombus. Two catheters were placed for bilateral emboli. Our protocol is based on the findings from the OTALYSE PE trial.^16^ Tissue plasminogen activator was infused at 1 mg/hour for 6 hours. A follow-up echocardiogram was then performed to assess improvement in the RV/LV ratio. In the absence of improvement, thrombolysis was continued for another 6 hours for a maximum total duration of 12 hours. Fibrinogen levels were monitored throughout treatment. Patients underwent repeat imaging with echocardiogram to evaluate RV/LV ratio at the conclusion of therapy.

Primary outcomes evaluated included ICU LOS in days, HLOS in days, 30-day survival, and 1-year survival. Secondary outcomes included absolute mean change in RV/LV from pre-procedure to post-procedure for each treatment group. For each individual patient, we measured the pre-to post-procedure change in RV/LV based on echocardiogram and then calculated the mean of the samples for each treatment type and reported standard deviations (SDs) from the mean. Other factors evaluated were: (1) presence of concomitant deep vein thrombosis (DVT) detected on preoperative lower extremity venous duplex; (2) preoperative elevated cardiac biomarkers including troponin >0.40 ng/mL, and BNP >100 pg/mL; (3) need for transfusion of packed red blood cells in the peri-procedural period; (4) occurrence of saddle PE; (5) prior history of DVT or PE; and (6) history of malignancy. Patients with high-risk PE (systemic hypotension <90 mmHg, use of vasopressors, or patients experiencing cardiac arrest), and pregnant patients were excluded. This study was granted a waiver of Institutional Review Board approval because it involved analysis of deidentified data and did not meet criteria for human subjects research.

### Statistical analysis

We presented continuous variables as means with SDs and 95% confidence intervals. Nonparametric data were presented as medians and interquartile ranges (IQRs) ranging from the 25th to 75th percentile range. We presented categorical variables as frequencies and percentages of the total sample, accounting for missing datapoints. We used two-sided unpaired Welch’s *t*-tests to compare independent groups not assumed to have equal variances, Mann-Whitney *U* tests to compare independent groups with nonparametric distribution, and paired *t*-tests and Wilcoxon signed-rank test to assess RV/LV ratios pre- and post-procedure. Z-tests were used to assess differences in proportions. Fisher’s exact tests were used to assess differences between categorical variables. A *P* value < .05 was regarded as significant. Statistical analysis was carried out using Jamovi, version 2.6 and Microsoft Excel.

## Results

### Clinical and periprocedural patient characteristics

One hundred twenty consecutive patients diagnosed with intermediate-risk PE by the previously described criteria were included in the study. One hundred patients underwent USAT (83%), and 20 patients underwent MT (17%). Baseline patient characteristics are presented in Table I. Factors, including sex, shortness of breath, saddle PE, cardiac biomarkers (troponin, BNP, both troponin and BNP, or neither troponin or BNP), and history of DVT, PE, or malignancy, did not differ between treatment groups. The MT group had both a greater proportion of patients with concomitant DVT (*P* = .033) and proportion of patients needing blood transfusion (*P* = .011) than the USAT group. Because packed red blood cell transfusion is an integer variable with a high proportion of zero values, transfusion is reported both as the proportion of patients requiring blood products and as mean ± SD and median (IQR) for completeness.

**Table I:** Patient characteristics

	USAT	MT	Total	*P*
Age, years	72.23 (12.6)	69.55 (15.7)	71.8 (13.1)	.479
Male sex	44 (44)	12 (60)	56 (47)	.225
Elevated biomarkers				.336
Troponin only	24 (24)	8 (40)	32 (27)	
BNP only	4 (4)	0 (0)	4 (3)	
Both	57 (57)	8 (40)	65 (54)	
None	15 (15)	4 (20)	19 (16)	
DVT	65 (65)	18 (90)	83 (69)	.033
SOB	80 (80)	16 (80)	96 (80)	1
Saddle	13 (13)	2 (10)	15 (13)	1
Transfusion PRBC	5 (5)	5 (25)	10 (8)	.011
Mean no. units transfused	0.1 (0.3)	0.7 (1.3)	0.2 (0.6)	.002
Median no. units transfused	0 (0)	0 (0.5)	0 (0)	
Prior DVT/PE	98 (98)		117 (98)	
DVT only	9 (9)	2 (11)	11 (9)	
PE only	4 (4)	0 (0)	4 (3)	
Both	3 (3)	3 (16)	6 (5)	
None	82 (84)	14 (74)	96 (82)	
Cancer history	19 (19)	4 (21)	23 (19)	1
Reason for MT				
Recent hemorrhagic stroke		2 (10)		
Active bleeding		3 (15)		
Recent surgery		5 (25)		
Thrombus location		10 (50)		

*BNP,* Brain natriuretic peptide; *DVT,* deep vein thrombosis; *MT,* mechanical thrombectomy; *PE,* pulmonary embolism; *PRBC,* packed red blood cells; *SOB,* shortness of breath; *USAT,* ultrasound-assisted catheter-directed thrombolysis.

Data are presented as number (%), mean (standard deviation), or median (interquartile range).

A significantly greater proportion of patients in the MT group required blood transfusion. Patients in the MT group had greater proportion of patients with concurrent DVT. Factors including cardiac biomarkers (troponin, BNP, both troponin and BNP, or neither troponin or BNP), SOB, saddle PE, history of DVT, PE, or malignancy, did not differ between treatment groups.

The Mann-Whitney *U* test was used to determine *P* values, due to nonparametric distribution, assuming unequal variances, and Fisher’s exact test was used to determine significance for categorical variables. Z-test was used to detect significance between proportions. *P* < .05 is significant.

Indications for MT included proximal thrombus location (50%) and contraindications to thrombolytic therapy including recent surgery (25%), active bleeding episode (15%), and recent hemorrhagic stroke (10%). Technical periprocedural success was achieved in all cases. For USAT, technical success was defined as successful placement of the thrombolysis catheter within the target pulmonary arteries with completion of the planned thrombolytic infusion without device-related complication or premature termination. For MT, technical success was defined as successful delivery and operation of the thrombectomy device with completion of clot extraction and restoration of pulmonary arterial flow without device failure or need for escalation to alternative therapy.

### Evaluation of preoperative RV/LV ratio across imaging modalities

As shown in Table II, CTA and echocardiography were performed preoperatively in 118 of 120 patients; 98 patients in the USAT group (98%) and 20 patients in the MT group (100%). Echocardiography alone was performed in one case. When evaluating RV/LV ratio preoperatively, we found a significant difference between preintervention RV/LV ratios measured by radiology on CTA and ratios reported by cardiology on echocardiography. Mean RV/LV ratio on CTA was 1.4 (SD, 0.4) and on echocardiography was 1.3 (SD, 0.2; *P* = .027).

**Table II:** Evaluation of right ventricle (*RV*)/left ventricle (*LV*) ratios between treatment groups (ultrasound-assisted catheter-directed thrombolysis [*USAT*] or mechanical thrombectomy [*MT*]) by imaging modality (computed tomography angiography [*CTA*] or echocardiogram [*echo*]) across the perioperative setting (preoperative or postoperative)

	USAT vs MT	No.	Missing	Mean (SD)	SE	95% confidence interval		Median	Min-max	*P*
						Lower	Upper			
RV/LV CTA preoperative	USAT	98	2	1.36 (0.31)	0.03	1.30	1.43	1.30	0.90-2.50	.173
	MT	20	0	1.60 (0.64)	0.14	1.30	1.90	1.50	1.00-4.00	
RV/LV echo preoperative	USAT	99	1	1.31 (0.23)	0.02	1.26	1.36	1.30	0.90-2.30	.069
	MT	20	0	1.32 (0.19)	0.04	1.22	1.41	1.35	0.90-1.60	
Postoperative echo RV/LV 6-12 hours	USAT	99	1	0.96 (0.17)	0.02	0.92	0.99	0.90	0.50-1.40	.072
	MT	10	10	1.07 (0.17)	0.05	0.95	1.19	1.05	0.80-1.40	
Preoperative to postoperative echo RV/LV ratio	USAT Pre-echo	99	1	1.31 (0.23)	0.02	1.26	1.36	1.30	0.90-2.30	<.001
	USAT Post-echo	99	1	0.96 (0.17)	0.02	0.92	0.99	0.90	0.50-1.40	
Preoperative to postoperative echo RV/LV ratio	MT Pre-echo	20	0	1.32 (0.19)	0.04	1.22	1.41	1.35	0.90-1.60	.006
	MT Post-echo	10	10	1.07 (0.17)	0.05	0.95	1.19	1.05	0.80-1.40	
Δ RV/LV ratio preoperaive-postoperative echo	USAT	100	0	0.36 (0.24)	0.02	0.32	0.41	0.30	0.10-1.80	.172
	MT	10	10	0.28 (0.17)	0.05	0.16	0.40	0.25	0.10-0.60	
% change RV/LV ratio	USAT	98	2	25.54 (12.33)	1.22	23.41	28.24	25.00	6.67-78.26	.189
	MT	10	10	20.21 (11.37)	3.60	12.07	28.35	17.69	6.67-42.86	
Imaging modality	CTA	118	2	1.40 (0.39)						.027
	Echo	120	0	1.31 (0.22)						
USAT	CTA	98	2	1.36 (0.31)						.173
	Echo	99	1	1.31 (0.23)						
MT	CTA	20	0	1.60 (0.64)						.069
	Echo	20	0	1.32 (0.19)						

*SD,* Standard deviation; *SE,* standard error.

Note: The confidence interval of the mean assumes sample means follow a t-distribution with N−1 degrees of freedom.

Delta (Δ) represents change from baseline preoperative value. There was no significant difference between USAT and MT treatment based on change in RV/LV ratios or percent change in RV/LV ratios from pre- to post-procedure as seen on Echo. CTA overestimated RV/LV ratio compared with Echo in the entire preoperative group.

Welch’s *t*-test was used to determine *P* values for independent samples, assuming unequal variances. Wilcoxon signed-rank test was used to determine significance between paired values. *P* < .05 is significant.

### Evaluation of changes in RV/LV ratios by echocardiography pre- and post-procedure across treatment modalities

Echocardiogram was conducted at 6 to 12 hours post-procedure to determine RV/LV ratio change. As shown in Table II, the USAT group had 99 patients (99%) imaged and the MT group had 10 patients imaged of 20 patients total (50%). There was significant improvement in the absolute mean RV/LV ratio from preoperative to postoperative echogram for both treatment groups (USAT, 1.31 to 0.96; *P* < .001; MT, 1.32 to 1.07; *P* = .006). There was no difference in absolute mean RV/LV ratio change (Δ) measured by echocardiography between USAT and MT groups from preoperative to postoperative (0.36; SD, 0.2 vs 0.28; SD, 0.2; *P* = .172) or percent change.

### HLOS, ICU LOS, and 30-day and 1-year survival rates of patients undergoing USAT and MT

As shown in Table III, MT patients had longer HLOS and ICU LOS than USAT patients, but these were not significant. Mean ICU LOS was 2.4 days; the median was 2.0 days (IQR, 2-3 days) for USAT and 2.6 days, and the median was 2.0 days (IQR, 2-3 days) for MT (*P* = .57). HLOS for patients undergoing USAT vs MT was 4.8 days; the median was 4.0 days (IQR, 3-6 days), and 5.7 days; the median was 4 days (IQR, 3-6 days; *P* = .95), respectively. Thirty-day survival was 98% in the USAT group and 100% in the MT patient group. At 1 year, survival was 96% in the USAT group and 90% in the MT group.

**Table III:** Hospital length of stay (*HLOS*) in days, intensive care unit (*ICU*) length of stay (*LOS*) in days, 30-day survival, and 1-year survival between ultrasound-assisted catheter-directed thrombolysis (*USAT*) and mechanical thrombectomy (*MT*) techniques

	USAT vs MT	No.	% of each group	Missing	Mean (SD)	SE	95% confidence interval		Median	IQR	25-75th percentile	Range	*P*
							Lower	Upper					
HLOS, days	USAT	100	100	0	4.75 (2.7)	0.27	4.21	5.29	4.00	3.00	3-6	1-17	.948
	MT	20	100	0	5.7 (4.9)	1.10	3.40	8.00	4.00	3.00	3-6	2-22	
ICU LOS, days	USAT	100	100	0	2.39 (1.1)	0.11	2.17	2.61	2.00	1.00	2-3	1-9	.566
	MT	20	100	0	2.6 (2.1)	0.47	1.62	3.58	2.00	1.25	2-3	1-10	
30-day survival	USAT	98	98	0									
	MT	20	100	0									
1-year survival	USAT	96	96	0									
	MT	18	90	0									

*IQR,* Interquartile range; *SD,* standard deviation; *SE,* standard error.

Mann-Whitney *U* test was used to determine *P* values due to nonparametric distribution, assuming unequal variances. *P* < .05 is significant.

## Discussion

Intermediate-risk PE represents a clinically challenging subgroup, characterized by hemodynamic stability with evidence of RV dysfunction and an associated mortality risk of approximately 5% to 15%.^6^^,^^23^ Although systemic thrombolysis can improve RV heart strain, its use in this population remains controversial due to increased bleeding risk and lack of clear mortality benefit over anticoagulation alone.^12^^,^^17^^,^^18^ Consequently, catheter-based interventions have emerged as attractive alternatives aimed at achieving hemodynamic improvement while mitigating bleeding complications.

In this single-center, PERT-driven cohort, we compared two commonly employed endovascular strategies—USAT and MT—for the management of intermediate-risk PE. Our findings demonstrate that both modalities are effective in improving RV function and are associated with excellent short- and intermediate-term outcomes, including low ICU LOS and HLOS, low perioperative mortality, and high 1-year survival.

### RV recovery and imaging considerations

Both USAT and MT resulted in significant reductions in RV/LV ratio, a well-established marker of RV strain and predictor of adverse outcomes.^13^ Notably, both USAT and MT groups experienced similar reduction in RV/LV ratio following treatment. These findings are consistent with prior MT trials, including FLARE and EXTRACT-PE, which demonstrated rapid RV improvement following aspiration thrombectomy with low rates of major bleeding.^23^, ^24^, ^25^

However, none of these studies compared MT with USAT.

Our analysis also highlights important considerations regarding evaluation of right heart strain. We observed that CTA significantly overestimated RV/LV ratios compared with echocardiography in the overall cohort. This discrepancy may be attributable to CTA’s static nature, patient positioning, timing of image acquisition, or inability to assess dynamic RV function. Furthermore, this could be due to overestimation of this ratio in patients with central PE or may reflect some hemodynamic improvement between the time of CTA and the echocardiogram, perhaps associated with fragmentation of a saddle thrombus. In contrast, echocardiography provides real-time functional assessment and remains central to risk stratification in intermediate-risk PE.^17^ This dynamic nature of echocardiography in detecting RV/LV ratio is the major reason we implemented echocardiography in intermediate-risk diagnosis in this consecutive patient cohort. In a larger cohort that included these patients along with patients who had both CTA and echocardiography but did not have intermediate-risk PE, we also found a tendency for CTA to overestimate RV/LV ratio. Together, we believe these observations support reliance on echocardiography—when feasible—in guiding interventional decision-making within PERT algorithms.

### Clinical outcomes and resource utilization

Along with similar early RV recovery between treatment groups in this intermediate-risk PE cohort, we found no differences in HLOS or ICU LOS, or 30-day or 1-year survival. Both treatment groups demonstrated equivalent ICU stays and HLOS, with no statistically significant differences between treatment modality. This finding contrasts with some prior reports suggesting shorter hospital stays with MT but aligns with other studies reporting comparable LOS between modalities.^26^^,^^27^ Differences across studies likely reflect institutional practice patterns, patient selection, thrombus burden, and availability of PERT-guided care.

MT was associated with a significantly higher need for packed red blood cell transfusion compared with USAT, as well as greater number of units transfused in patients requiring transfusion. This finding is clinically relevant and may reflect intraprocedural blood loss, procedural complexity, or selection bias. MT was preferentially used in patients with central clot burden or contraindications to thrombolysis. This observation underscores the importance of individualized treatment selection, particularly in patients with anemia, bleeding risk, or contraindications to transfusion. Patients in the MT cohort had greater incidence of DVT than the USAT group. This finding is unexplained but may suggest that patients selected for MT were more severe at baseline or presented with higher clot burden. We cannot exclude the possibility that patients in the USAT group had unrecognized pelvic DVT or complete embolization of their extremity DVT, although there is no evidence to support this.

### Role of PERT and treatment selection

PERT programs were developed to facilitate rapid stratification and multidisciplinary PERT decision-making, enabling individualized selection of advanced therapies based on patient characteristics, clot location, and bleeding risk.^24^^,^^25^^,^^28^^,^^29^ Despite widespread adoption of this approach, real-world outcome data comparing these strategies within a PERT framework are sparse. Our results reinforce the value of a multidisciplinary PERT model in managing intermediate-risk PE. The excellent outcomes observed in both groups likely reflect timely diagnosis, careful risk stratification, and identification of contraindications to thrombolytic therapy. In our practice, MT is favored when the majority of thrombus is centrally located within the main pulmonary arteries, if severe physiologic abnormalities are present that require rapid reduction in clot burden, or when thrombolysis is contraindicated, whereas we use USAT when clot is primarily located in segmental or subsegmental branches without contraindication to thrombolysis. We believe the excellent short- and intermediate-term results in this group of patients reflect the value of a PERT team and support our current clinical practice.

### Limitations

This study has several limitations. Its retrospective design introduces potential for selection bias, given the nonrandomized allocation of treatment modalities and the imbalance in group sizes favoring USAT. Our aim was not to prove superiority of one technique but to analyze our results with our current selection algorithm. Multiple imaging modalities (CTA and echocardiogram) were used to measure RV/LV ratios and were compared against each other. However, when calculating change in RV/LV ratio, we used only the preoperative and postoperative echocardiograms. It is possible that gated CTA imaging and alternative functional parameters might give similar results to echocardiography, and additional research in this area is warranted. Not every patient in the MT cohort had postoperative imaging, leading to decreased numbers in the sample compared with the USAT cohort. This is most likely because postoperative imaging was mandatory in all USAT patients to determine the need for ongoing thrombolysis, whereas postoperative imaging in the MT group was more likely driven by clinical indications. If anything, we can postulate that the MT patients who were not imaged post procedure had as good or even better clinical outcome and were more likely to have even lower RV/LV ratio than those who were imaged, although this remains speculation. There is limited generalizability of these study findings, given that these patients were from a single, high-volume PE center. Finally, although we assessed short- and intermediate-term outcomes, longer-term functional and quality of life outcomes were not evaluated.

## Conclusions

In this single-institution, PERT-guided cohort of patients with intermediate-risk PE, both USAT and MT were safe and effective, producing significant improvement in RV function and excellent short- and mid-term survival. Overall clinical outcomes and RV recovery were comparable between modalities. MT demanded increased blood transfusion compared with USAT, and more patients in this cohort had evidence of lower extremity DVT. These findings support a tailored, anatomy- and patient-specific approach to intervention and underscore the importance of echocardiography in risk stratification. Further multicenter, prospective studies are needed to better define long-term outcomes and optimize treatment algorithms for this complex patient population.

Taken together, our experience reflects the outcomes that can be achieved with intervention for intermediate-risk PE. These findings underscore the importance of continuous development of PERT teams and algorithms in the complex management of pulmonary embolism.

## Author Contributions

Conception and design: CH, MW, MS, LH, JS, JR, JJR

Analysis and interpretation: CH, MW, MS, JR

Data collection: CH, MW, MS, DM

Writing the article: CH, MW, MS, DM, JR

Critical revision of the article: CH, MW, LH, JS, JR, JJR

Final approval of the article: CH, MW, MS, DM, LH, JS, JR, JJR

Statistical analysis: CH, JR

Obtained funding: Not applicable

Overall responsibility: CH

## Funding

None.

## Disclosures

None.
